# Vascular multiple sclerosis: addressing the pathogenesis, genetics, pro-angiogenic factors, and vascular abnormalities, along with the role of vascular intervention

**DOI:** 10.1097/MS9.0000000000001177

**Published:** 2023-08-14

**Authors:** Priyadarshi Prajjwal, Anagha Shree, Soumyajit Das, Pugazhendi Inban, Shankhaneel Ghosh, Arihant Senthil, Jaiprakash Gurav, Mrinmoy Kundu, Mohammed Dheyaa Marsool Marsool, Srikanth Gadam, Ali Dheyaa Marsool Marsoo, Neel Vora, Omniat Amir Hussin

**Affiliations:** a Department of Neurology, Bharati Vidyapeeth University Medical College; b Armed Forces Medical College, Pune; c Institute of Medical Sciences and SUM Hospital, Bhubaneswar; d Internal Medicine, Government Medical College, Omandurar, Chennai; e University College of Medical Sciences, Delhi; f SGT Medical College Hospital and Research Institute, Gurgaon; g Internal Medicine, B.J. Medical College, Ahmedabad, India; h Internal Medicine, Mayo Clinic, Rochester, Minnesota, USA; i Al-Kindy College of Medicine, University of Baghdad, Baghdad, Iraq; j Internal Medicine, Al-Manhal Academy, Khartoum, Sudan

**Keywords:** cerebral venous insufficiency, endothelial dysfunction, inflammation, vascular multiple sclerosis

## Abstract

Dysfunction in the epithelium, breakdown of the blood–brain barrier, and consequent leukocyte and T-cell infiltration into the central nervous system define Vascular Multiple Sclerosis. Multiple sclerosis (MS) affects around 2.5 million individuals worldwide, is the leading cause of neurological impairment in young adults, and can have a variety of progressions and consequences. Despite significant discoveries in immunology and molecular biology, the root cause of MS is still not fully understood, as do the immunological triggers and causative pathways. Recent research into vascular anomalies associated with MS suggests that a vascular component may be pivotal to the etiology of MS, and there can be actually a completely new entity in the already available classification of MS, which can be called ‘vascular multiple sclerosis’. Unlike the usual other causes of MS, vascular MS is not dependent on autoimmune pathophysiologic mechanisms, instead, it is caused due to the blood vessels pathology. This review aims to thoroughly analyze existing information and updates about the scattered available findings of genetics, pro-angiogenetic factors, and vascular abnormalities in this important spectrum, the vascular facets of MS.

## Introduction

Highlights

- This review introduces the concept of ‘vascular multiple sclerosis’, highlighting its distinct characteristics characterized by endothelial dysfunction, blood–brain barrier (BBB) disruption, and immune cell infiltration into the central nervous system.
- The review sheds light on recent research suggesting that vascular anomalies play a crucial role in the etiology of multiple sclerosis (MS). It proposes the existence of a new entity, ‘vascular multiple sclerosis’, which is not dependent on autoimmune mechanisms but rather on pathology related to blood vessels.
- Despite significant advances in immunology and molecular biology, the root cause of MS and its immunological triggers remain unclear. This review aims to fill existing knowledge gaps by thoroughly analyzing available information on genetics, pro-angiogenetic factors, and vascular abnormalities associated with vascular MS.
- By focusing on the vascular facets of MS, this review contributes to a better understanding of the disease and its diverse advancements and outcomes.
- The review extensively analyzes scattered findings, consolidating information on vascular abnormalities in MS. Examining the role of endothelial dysfunction, BBB disruption, and immune cell infiltration offers insights into the complex interplay between vascular pathology and the development of vascular MS.

The chronic condition, multiple sclerosis (MS), that affects the central nervous system (CNS) is characterized by inflammation and damage to the protective myelin sheath around nerve fibers. The autoimmune theory of the disease has been widely accepted due to a significant amount of evidence supporting it^1–3^. But, recent research into vascular anomalies associated with MS suggests that a vascular component plays an important role in the development of MS and can lead to vascular-specific signs and symptoms, thereby contributing to the rare subtype of MS, vascular MS. ‘Vascular MS’ is an emerging interest among the researchers and it could well be added as one of the various types of MS in the near future depending upon the novelty and wide difference in its pathogenesis and immunology from the usual relapsing–remitting, primary-progressive, and secondary-progressive types of MS.

Essentially, various types of vascular anomalies have been linked to MS, including disruption of the blood–brain barrier (BBB)^4^, endothelial cell dysfunction^5^, an elevated risk for ischemic disease due to hypoxia and inflammation^6^, decreased brain blood flow^7^, and poor drainage of blood from veins^8^. Studies have demonstrated that the alterations in the BBB and the transmigration of inflammatory cells and leukocytes from the peripheral circulation across the endothelial barrier are among the initial and most prevalent anomalies detected in the brains of persons with MS. It is believed that breaching the BBB process is a critical component in the formation of demyelinating lesions^9^. Once these leukocytes become active in the CNS, they trigger a destructive wave that results in the loss of both myelin and oligodendrocytes, as well as neuronal degeneration. Numerous clinical and scientific findings suggest that stress and apoptosis of endothelial cells are defining features of MS^10^.

Hypoxia has been linked to MS and research suggests that a decrease in the overall metabolic rate in individuals with MS may indicate that ischemia is not limited to lesions, but instead affects the CNS as a whole. The hypoxic response pathway is closely related to the inflammatory pathway and involves a group of oxygen-sensing molecules called prolyl hydroxylases (PHDs)^11^.

A study by Laupacis *et al*.^12^ found that the pathogenesis of MS involves aberrations in the direction and pathway of blood flow in the cerebral veins, resulting in iron accumulating in the brain, and this causes an autoimmune reaction to be triggered. One such condition is chronic cerebrospinal venous insufficiency (CCSVI), which is a long-lasting condition characterized by insufficient drainage of blood from the CNS^13^. The presence of CCSVI, characterized by anomalies in the anatomy and flow of internal jugular, deep cerebral, vertebral, and azygous veins, is more prevalent among individuals with MS compared to those without the disease^12^.

This review aims to thoroughly analyze the existing information about the connection between vascular comorbidities and MS. Based on the available evidence, there appears to be a higher likelihood of developing MS among individuals with vascular abnormalities.

## Risk factors for vascular MS

### Hypertension

Hypertension is a common vascular risk factor that increases the risk of developing chronic diseases, including vascular MS^14–16^ (Table 1). Hypertension affects the blood vessels and disrupts the BBB, which functions as a physiological partition that separates the CNS from the peripheral tissues. This barrier is made up of densely packed cells that keep noxious substances out of the brain and spinal cord. The breach of the BBB allows for the infiltration of immune cells into the brain, leading to the onset of inflammation, potentially leading to the development of vascular anomalies triggering the vascular MS.

**Table 1:** Different risk factors implemented in the pathogenesis of vascular multiple sclerosis and their proposed mechanism.

Risk factor	Description	Mechanism
Smoking	Cigarette smoking	May damage blood vessels and increase inflammation, which can lead to vascular MS
Hypertension	Elevated blood pressure	Damages blood vessels and disrupts the blood–brain barrier, which may increase the risk of developing vascular MS
Obesity	Excess body weight, often defined as a body mass index (BMI) of 30 or higher	Studies have shown that excess body weight and obesity during adolescence increase the risk of developing vascular and usual MS later in life
Diabetes	Impairment of the ability of the body to produce or use insulin effectively	Individuals with type 1 diabetes have been shown to have a higher risk of developing MS in comparison to the general population
Dyslipidemia	Elevated concentrations of lipids in the bloodstream, encompass heightened levels of cholesterol and triglycerides	This condition can increase the risk of developing vascular MS by damaging blood vessels and disrupting the blood–brain barrier
Vitamin D deficiency	Low vitamin D levels in the blood	This may contribute to an imbalance in the immune system and the development of autoimmunity, as well as a disruption of the blood–brain barrier
Inactivity	Sedentary lifestyle with little or no physical activity	May increase inflammation and contribute to the development of vascular MS

The existing literature on the incidence of hypertension among individuals with MS presents conflicting findings. Several investigations have reported comparable or reduced occurrences of hypertension among individuals with MS in comparison to the control group^17–19^. Kang *et al*.^20^ reported a higher incidence of hypertension among individuals with MS (*n*=898) in comparison to the control group.

### Cigarette smoking

Smoking is a well-known and significant risk factor for vascular facets of MS. A large body of research has consistently shown that individuals who engage in smoking behavior are at an increased likelihood of developing MS in comparison to those who do not smoke^21–23^. Furthermore, smoking has been linked to an increase in the severity and progression of MS symptoms^22^. It is hypothesized that smoking could potentially play a role in the pathogenesis of MS by inducing vascular damage and compromising BBB integrity. This may lead to the infiltration of immune cells and pro-inflammatory molecules into the CNS, ultimately resulting in tissue damage and clinical manifestations of the disease. Studies have demonstrated that smoking can elevate oxidative stress and inflammation levels, which are believed to contribute to the pathogenesis of this disorder^24^.

### Obesity

Obesity is one of the major risk factors that can lead to vascular MS^25^. The correlation between being overweight or obese during adolescence and an elevated likelihood of developing MS in later stages of life has been established through scientific research^26,27^. Several studies conducted during childhood and adolescence found a link between body mass index (BMI) and the risk of MS development^28^. One another study reported a decreased obesity prevalence among individuals with MS in comparison to their counterparts without the condition^29,30^.

Obesity is also linked to other vascular risk factors that have been linked to an increased risk of MS, such as hypertension, dyslipidemia, and insulin resistance^31^. The available evidence suggests that obesity could potentially exacerbate pre-existing symptoms, such as fatigue, disability, and depression in individuals with MS^32,33^. Interestingly, there is enough evidence to suggest that losing weight may be beneficial in managing MS symptoms. A recent study discovered that a low-calorie diet followed by a 6-month weight maintenance period improved fatigue, life quality, and disability in patients living with MS who were overweight or obese^34^.

### Diabetes

There exists a correlation between diabetes, particularly type 1 diabetes (T1D), as a vascular risk factor in the development of MS^35^. Type 1 diabetes mellitus is a pathological condition characterized by an aberrant immune response that leads to the destruction of pancreatic beta cells, which are responsible for the production of insulin^36^. Elevated blood glucose levels can lead to vascular impairment and elevate the likelihood of vascular MS onset^37^.

A higher risk has been identified in individuals with T1D for the development of MS than in the general population. Furthermore, a recent study discovered that people with T1D who also had high levels of antibodies against a protein known as myelin oligodendrocyte glycoprotein (MOG) had a significantly higher MS risk than those who did not^38^. In a cohort study, which encompassed 98% of the pediatric diabetic population under 21 years of age in Austria and Germany, the risk ratio (RR) of MS co-occurrence was investigated. The study revealed that the RR for MS in T1D was three to almost five times greater than in the non-diabetic population^39,40^.

### Dyslipidemia

Dyslipidemia is a medical condition that is distinguished by atypical lipid concentrations in the bloodstream, encompassing elevated levels of cholesterol and triglycerides^41^. Dyslipidemia can increase the risk of developing the vascular spectrum of MS by damaging blood vessels and disrupting the BBB, increasing the likelihood of immune cells entering the CNS and causing inflammation^42^. The findings of a case–control study indicated that individuals with MS exhibited elevated levels of total and low-density lipoprotein (LDL) cholesterol in comparison to the control group^43–45^.

### Vitamin D deficiency

The association between the vascular spectrum of MS and vitamin D deficiency has been established in several studies^46^. Vitamin D is a crucial element for maintaining optimal bone health and plays a significant role in regulating the metabolism of both phosphorus and calcium^47^. It does, however, have immunomodulatory properties and is involved in immune function regulation^48–50^.

Several hypotheses have attempted to explain the link between vitamin D deficiency and vascular MS. One possibility is that low vitamin D levels contribute to immune dysfunction, resulting in autoimmunity and, eventually, MS^48^. Vitamin D has been shown to have anti-inflammatory properties, and low levels may increase inflammation and damage to the myelin sheath surrounding nerves, which is a symptom of vascular MS^51^.

Another proposed mechanism is that vitamin D may help to maintain the BBB’s integrity^52^. This notion is supported by studies showing that vitamin D deficiency resulted in greater cortical and striatal infarction and behavioral impairments^52^, but its specific role in increasing stroke-induced BBB permeability needed more investigation. Notwithstanding, the correlation between vitamin D and MS remains incompletely comprehended, and further investigation is required to establish the precise underlying mechanisms.

### Sedentary lifestyle

The state of being sedentary or engaging in minimal physical activity is a widely recognized risk factor for various chronic illnesses such as diabetes, cardiovascular disease, and obesity^53^. Physical activity may contribute to the development and progression of vascular and usual MS, according to emerging shreds of evidence^54,55^.

Individuals with MS who engage in regular physical activity have better cardiovascular health, improved mood, and higher levels of overall functioning than those who do not^56^. Furthermore, physical activity may help reduce inflammation, improve immune function, and promote nerve cell growth and survival, all of which may be beneficial in MS^57^.

A protective effect against MS may be conferred by physical activity, which can upregulate neurotrophic factors synthesis, a protein class that facilitates the growth and survival of neurons. Research studies have demonstrated that physical exercise has the potential to elevate brain-derived neurotrophic factor (BDNF) levels. This protein has been associated with neuroprotection and nerve regeneration in individuals with MS^58^.

## Pathophysiology of vascular MS

### Endothelial microparticles

**Figure 1:**
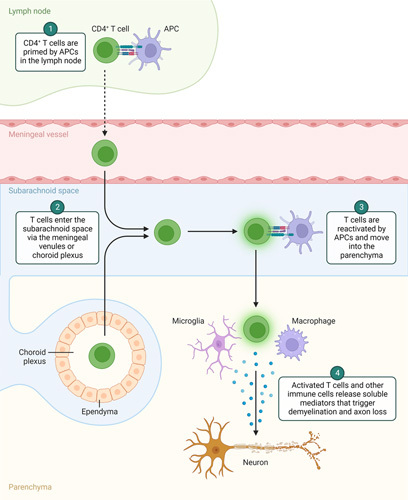
A summary of the mechanisms of the autoreactive T-cell response and the resultant autoimmune disease of multiple sclerosis in the central nervous system. APCs, antigen-presenting cells.

Endothelial microparticles (EMPs) are minute vesicles that are discharged from the plasma membranes of endothelial cells when these cells are activated, damaged, or undergo apoptosis. These particles are recognized to have a crucial role in various biological processes, such as inflammation, vascular injury, angiogenesis, and thrombosis. EMPs have been associated with the disease pathophysiology of various conditions, including atherosclerosis and hematological and systemic inflammatory diseases, including the vascular spectrum of MS (Fig. 1)^59^.

Endothelial disruption, a hallmark process in the pathophysiology of MS, has been linked to EMPs. The effect of purified microparticles (MPs) on endothelial barriers was assessed by Marcos-Ramiro *et al*. using electric cell-substrate impedance sensing. MPs from samples of patients with relapsing–remitting MS (RRMS) resulted in more pronounced disruption of endothelial barriers *in vitro* when compared to those acquired from healthy donors or individuals with clinically isolated syndrome (CIS)^60^. Minagar *et al*.^10^ suggested the involvement of plasma factors in endothelial dysfunction, as evidenced by the MS plasma-induced release of CD31+ and CD51+ EMPs from microvascular endothelial cell (MVEC) culture in-vitro studies^10^.

Following endothelial dysfunction and BBB disruption, the transmigration of activated monocytes and T cells is an established process in MS pathophysiology. EMPs have been implicated in this scenario too. Jy *et al*.^61^ found that EMPs bound preferentially to monocytes as compared to other leukocytes, and observed that during periods of exacerbation in MS patients, there was a significant increase in EMP–monocyte conjugate (EMP–MoC) levels compared to remissions and controls, suggesting the possible role of these conjugates as markers of disease activity. Furthermore, the EMPs in these conjugates activated their bound monocytes and facilitated their trans-endothelial migration (TEMIG). The addition of MS plasma increased the ability of monocytes to migrate through cerebral endothelial cells (CECs) and pre-treating monocytes with EMPs also enhanced their migration in an in-vitro model. The findings indicate that EMP–monocyte complexes exhibit a greater propensity for TEMIG in comparison to monocytes in isolation. Based on the results obtained, it can be inferred that extracellular vesicles known as EMPs play a crucial role in the development of MS.

#### Biomarkers based on EMPs for vascular MS

EMPs could serve as potential biomarkers for MS. Marcos-Ramiro *et al*.^60^ reported alterations in the levels of circulating MPs in individuals suffering from MS as compared to healthy subjects. The researchers employed flow cytometry to examine the plasma collected from 95 patients exhibiting various clinical forms of MS, as well as 49 healthy individuals, to detect the existence of platelet-derived and endothelium-derived MPs. The findings indicate that there is a notable increase in the levels of EMPs among individuals diagnosed with MS or CIS in comparison to the control subjects.

Moreover, EMPs expressing distinct protein markers are differentially elevated in various stages of MS. Minagar *et al*.^10^ assayed the plasma of patients for EMPs expressing CD31 (PECAM-1) and CD51 and found that during periods of exacerbation in MS patients, there was a marked increase in the levels of CD31+ EMPs, but this was not observed during remission. In contrast, plasma CD51+ EMPs were consistently elevated, irrespective of whether the patients were in exacerbation or remission, suggesting that these may reflect chronic endothelial injury. Similarly, Zinger *et al*.^62^ found that there is a significant increase in EMP levels among MS patients who have not undergone treatment, as compared to both healthy individuals and MS patients who have received Fingolimod treatment.

There exists a correlation between certain MP species and the presence of depositions of iron in subcortical deep gray matter structures. Alexander *et al*.^63^ examined the sera of patients for three types of MPs [CD31+/PECAM-1, CD51+CD61+ (αV–β3), and CD54+ (ICAM-1)], and cross-referenced these with traditional magnetic resonance imaging (MRI) and newer MRI techniques that evaluate iron levels using susceptibility-weighted imaging (SWI)-filtered phase. The findings indicate that the occurrence of different MP species differed among individuals who were in good health, those who had RRMS, and those who had secondary-progressive MS (SPMS). Furthermore, a multitude of MP variants (specifically those expressing CD31, CD51, CD61, and CD54) exhibited a significant correlation with conventional MRI and SWI characteristics of MS. Hence, it is suggested that analyzing MP profiles has the potential to serve as an additional, convenient, non-invasive method to MRI for monitoring disease activity in MS.

#### Therapeutic advances in EMP and Fingolimod to manage vascular MS

Fingolimod, a drug used in the management of MS, may function, in part, by altering EMP mechanisms. Zinger *et al*.^62^ exposed human brain endothelial cells (HBECs) to varying concentrations of tumor necrosis factor and measured the release of MPs in the presence and absence of Fingolimod. In the presence of Fingolimod, TNF-induced EMP release was shown to be greatly reduced. This suggests that a reduction in EMP release may be one of the mechanisms by which Fingolimod prevents exacerbations and slows the progression of MS.

In a study by Sheremata *et al*.^64^, IFN-β1a therapy given to RRMS patients resulted in a significant reduction of plasma levels of CD31+ EMPs, suggesting the potential for plasma CD31+ EMP levels to be used as a marker for monitoring response to IFN-β1a.

Jimenez *et al*.^65^ observed that the TEMIG of monocytes and EMP–MoCs was inhibited by IFN-β1b. Pre-incubation of brain MVEC (BMVEC) cultures with IFN-β1b resulted in a reduction of TEMIG of monocytes and EMP–MoC complexes, as assessed by flow cytometry. These findings suggest a novel mechanism for the protective role of IFN-β1b in MS patients. Therefore, these findings suggest that the identified targets hold promise as viable therapeutic options for managing MS.

### Pathophysiology of endothelial junctional abnormalities in vascular MS

As mentioned earlier, in vascular MS, the permeability of the BBB is increased, and this is connected to the development of lesions as well as problems with the tight junctions (TJs) in the microvasculature. In their investigation, Kirk *et al*.^66^ aimed to study TJ disruption in different subtypes of MS patients, and their association with BBB leakage. The research entailed the examination of cryosections of plaques and normal-appearing white matter obtained from individuals diagnosed with MS, as well as those with other neurological conditions, and compared them to healthy control samples. Tissue samples from these groups were evaluated for the presence of the protein zonula occludens-1 (ZO-1), which is associated with the TJ, and its relationship to fibrin leakage was assessed.

The study revealed that TJ abnormalities had notable variations in frequency across MS lesion types and white matter controls. Additionally, the investigators observed that these irregularities were more prominent in active MS lesions. They concluded that the disintegration of TJs in MS plays a role in promoting BBB leakage. Also, these findings were consistent across different vessel sizes, suggesting the involvement of soluble factors that can diffuse across vessels in mediating TJ injury.

TJ proteins, specifically occludin, and VE-cadherin, have been implicated in BBB disruption in MS. The expression of these proteins is likely affected by circulating inflammatory cytokines like TNF-α and IFN-γ. When Minagar *et al*.^67^ exposed cultured endothelium cells to sera from MS patients who were in exacerbation, remission, and healthy controls, they saw that the expression of occludin and VE-cadherin was decreased, with a more significant reduction noticed for occludin compared to VE-cadherin. The authors concluded that high levels of cytokines that are pro-inflammatory in the serum during exacerbations led to the suppression of VE-cadherin and occludin. In another study, this was found to be true for IFN-γ also^68^. The role of occludin in this scenario is further evidenced by the findings of Morgan *et al*.^69^, who examined proteins in rats with and without experimental autoimmune encephalomyelitis (EAE), a model for MS, in their spinal cords. Occludin was found to be dephosphorylated in EAE rats, and this dephosphorylation occurred just before changes in BBB permeability, suggesting that this process may be involved in regulating the response of the BBB to inflammation.

Research toward using occludin and related proteins like ZO-1 as biomarkers for disease activity in MS has been inconclusive and further studies with larger cohorts are required^70^.

Padden *et al*.^71^ studied the expression of other TJ proteins in MS. Vascular endothelial junction-associated molecule (VE-JAM), platelet F11 receptor (P-F11R), and β-catenin expression levels were evaluated in blood vessels obtained from lesions that are active in MS, dormant lesions, normal-appearing white matter, as well as control white matter. P-F11R expression was shown to be aberrant with greater frequency than other proteins in active lesions of white matter. On the other hand, in both normal and MS tissues, VE-JAM was discovered to be strongly expressed in a small number of major blood vessels. Additionally, β-catenin was expressed regularly across all categories of MS and control tissue. The authors proposed that changes in the expression of P-F11R might impact both the tightness of junctions and leukocyte trafficking.

### Chronic cerebral venous insufficiency in vascular MS

A significant correlation was found recently between vascular facets of MS and a condition called chronic cerebrospinal venous insufficiency (CCSVI). CCSVI refers to a vascular condition that impacts the primary extracranial cerebrospinal venous pathways, resulting in abnormal blood outflow in MS patients^8^.

CCSVI initiates a sequence of events caused by abnormal venous hemodynamics that disrupts microcirculation. This leads to the leakage of red blood cells (RBCs), which is the primary source of iron accumulation. In MS patients with CCSVI, the obstruction of extracranial veins is believed to overwhelm the microcirculation in the brain, as confirmed by the presence of RBCs outside the blood vessels in brain plaques located near the veins^72^.

Histopathological studies have revealed a unique pattern of iron accumulation in MS patients, with the iron stores consistently surrounding the walls of veins. The iron deposits take the form of hemosiderin buildup and structures similar to ferritin found within macrophages. Interestingly, this pattern is similar to the perivenous iron accumulation commonly seen in peripheral venous disease^73^. Various other studies suggest that venous abnormalities can be found in the internal jugular veins, vertebral veins, and azygous vein^72,74^ and can be detected through selective venography, extracranial venous echo-color Doppler (ECD), and magnetic resonance venography (MRV) with varying degrees of accuracy^75^. The diagnosis of CCSVI relies on evaluating a Doppler sonography protocol that utilizes both extracranial and transcranial VH criteria. Various connections have been found between abnormal hemodynamic MRI results, clinical outcomes, and CCSVI in MS patients^76^.

A study was conducted on a group of 65 patients with CCSVI who had undergone selective venography which allowed them to identify venous obstructions and treat them with balloon angioplasty during the diagnostic evaluation. Patients were monitored for 18 months to evaluate their vascular and neurological outcomes related to MS^72^.

### Role of genetics in MS

It has been well established that there is a genetic predisposition to developing vascular facets of MS. Studies on families have shown varying degrees of heritability, with a recent large cohort in Sweden demonstrating a significantly increased risk among siblings^77^. Through genome-wide association studies (GWAS), more than 100 distinct loci have been identified as potential contributors to the development of MS, most of which have been linked to immune function. While these genes are crucial for the pathological immune response in vascular MS, they may also play independent roles in the early dysfunction of the BBB^78^. It is worth noting that there exists a certain degree of genetic correlation between MS and cardiovascular disease, indicating a potential genetic impact on the vasculature or endothelium, contributing to the vascular manifestations^79^.

The results indicate that transcription factors and regulators have a significant impact on connecting changes in vascular function with MS pathology. Additionally, these findings provide evidence for the presence of pleiotropic loci among MS and cardiovascular comorbidities. LEF1 and SMARCA4, both members of the WNT/beta-catenin pathway, have been linked to vascular remodeling, post-stroke angiogenesis, and cardio-cerebral-peripheral vascular diseases^80,81^.

The genes SMARCA4, AFF1, and NR1D1/Rev-Erbα are interconnected with immunity, lipid metabolism, and vascular function. Additionally, SMARCA4 and AFF1 have been observed to correlate with plasma lipid levels. This information is supported by previous research^80^.

Both ischemic stroke and MS have the CAMKG2/CaMKIIγ protein implicated. Vascular smooth muscle proliferation and remodeling are both functions of this protein. The study suggests that CD86 and BATF are associated with both MS and cardiovascular disease risk factors. Specifically, CD86 is found to be responsible for driving the atherogenic process, while BATF is linked to HDL (high-density lipoprotein) cholesterol and systolic blood pressure. The study has identified the 5′UTR CD86 rs11575853 as a significant factor associated with decreased expression of IQCB1, a gene that plays a crucial role in retinal diseases and may be linked to neurodegeneration.

The venous tissue of MS patients, particularly in the internal jugular vein, has shown differential expression of several genes^82^. Among these genes, 16 were previously identified by GWAS, while seven genes were not previously identified but have functional interaction with MS candidates. The majority of these genes are associated with vascular or neuronal traits/diseases, along with MS, and nine of them encode transcriptional factors/regulators. Additionally, a set of eight genes have been identified as having a correlation with plasma lipid levels, providing further evidence for the connection between lipids and immune-mediated disorders^83^.

### Pro-angiogenetic factors in vascular MS

A series of orchestrated modifications in the endothelial cells characterizes the process of angiogenesis within the CNS, ultimately resulting in the development of a highly impermeable barrier. This barrier has several features that selectively allow certain molecules to pass through, thereby maintaining CNS homeostasis and preventing unwanted substances from entering the tissue^84^. The mechanisms and cell types involved in regulating the formation of this barrier during developmental angiogenesis have been described. The occurrence of angiogenesis has been noted in CNS disorders that exhibit impaired barrier function, including MS and stroke.

Angiogenesis in MS lesions leading to the vascular manifestations is influenced by several factors, including the hypoxic environment present in active lesions in patients with MS^85^. As a result of diverse alterations in the surrounding pathological milieu, the hypoxic insult occurs. For instance, active MS lesions express hypoxia-inducible factor 1-alpha (HIF-1α) to a great extent. Upregulating the expression of Na+ channels that are prone to leakage is the way these axons that have lost their myelin sheath compensate for the inefficient propagation of saltatory action potentials. This results in an increased demand for energy in the affected tissue. Furthermore, there is an elevated inducible nitric oxide synthase (NOs) level exhibited by the active lesions in MS, resulting in heightened NO concentrations that impede mitochondrial respiration^86^. The augmented energy requirements and immune cell infiltration, in conjunction with paracrine signaling, culminating in a state of hypoxia characterized by an inadequate supply of oxygen relative to demand, thereby stimulating the formation of new blood vessels.

Given that hypoxia-like conditions play a crucial role in the pathology of vascular MS, it is plausible that molecules that act as angiogenic signals in ischemic diseases such as stroke or tumor-induced angiogenesis could potentially stimulate angiogenesis in it. Studies have found high levels of HIF-1 and vascular endothelial growth factor (VEGF), which are crucial factors for VEGF-induced angiogenesis, in active lesions of MS patients^87^. The serum of MS patients exhibits elevated levels of Endothelin-1 and angiopoietin-2, both of which augment the angiogenic properties of VEGF^88^. The presence of NOs has been found to play a role in the development of new blood vessels in inflammatory diseases. In patients with MS, there is an increase in NOs levels, which is associated with inflammation and MRI markers indicating the progression of the disease. The involvement of fibroblast growth factor (FGF) in angiogenesis in MS has been reported, which can also contribute to the vascular manifestations. Transcript analysis has revealed increased expression of FGF-12 and FGF-2 homolog in MS lesions, and MS patients have shown elevated serum FGF levels^89^.

Alongside hypoxia-related factors, certain molecules implicated in the pathogenesis of vascular MS possess properties that modulate angiogenesis. The presence of TNF-α and INF-γ, which are inflammatory cytokines found in MS, has been observed to potentially stimulate angiogenesis^90^. Several matrix metalloproteinases (MMPs) have been found to play a role in promoting inflammation and breaking down the extracellular matrix, which aids in the growth of new blood vessels during the progression of MS^91^. Additional investigation is necessary to ascertain the precise impacts of these variables on neo-angiogenesis as opposed to inflammation within the framework of MS pathology.

### Vascular abnormalities

The relationship between demyelination and the impairment of MS is significantly influenced by the vascular system. Prior research has focused on exploring four principal vascular anomalies linked to MS, which include: (i) inadequate delivery of oxygen to brain tissues, a condition referred to as hypoxia^92^, (ii) reduced cerebral blood flow (CBF)^92^, (iii) malfunctioning of the BBB, and (iv) the infiltration of metabolites, including fibrin, into the brain parenchyma is a consequence of the aforementioned factors. Additionally, compromised drainage of harmful waste products from the brain through the jugular veins is observed. This is supported by previous research^93,94^.

#### Hypoxia and inflammation

The association between hypoxia and MS has been established for a considerable period^95^. Lesions classified as Type III in MS brains exhibit similar histological characteristics as lesions resulting from lack of oxygen or blood supply, which tend to develop in regions of the brain that are more vulnerable to hypoxia^96^. According to a previous study^11^, it has been observed that there is a widespread presence of diffuse hypoxia in MS, and this has led to the proposal of an interdependent relationship between hypoxia and inflammation in MS that fuels the progression of the disease and vascular facets of it. Multiple studies have employed MRI to demonstrate that individuals with MS exhibit a lower amount of CBF in comparison to those without the disease, and this reduction is most likely a consequence of impaired vasodilation^97^. The correlation between impaired vasodilation, decreased CBF, and gray matter loss, along with cognitive impairment, indicates that inadequate blood supply (and consequent hypoxic stress) could have a significant contribution toward neurodegeneration in MS^98,99^.

**Figure 2:**
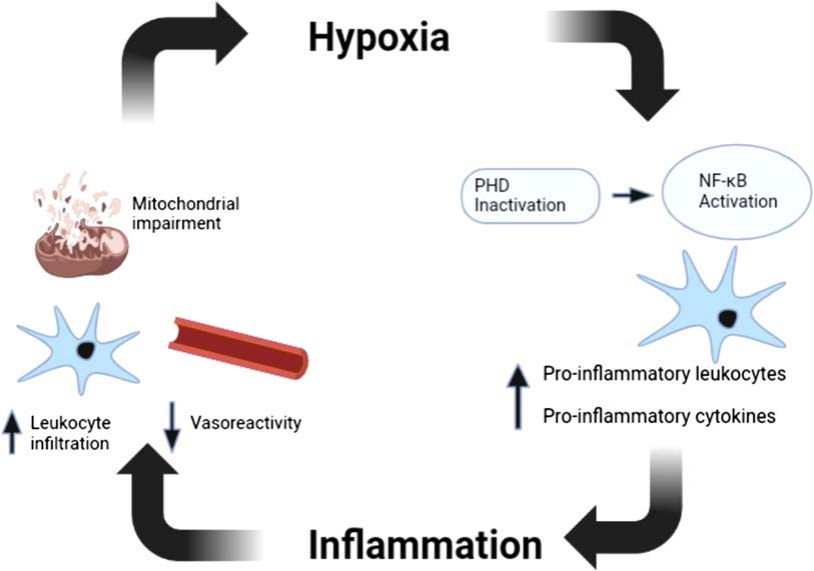
The positive feedback loop between hypoxia and inflammation (original figure, created from Biorender, modified from Figure by Yang *et al*.^11^). PHD, prolyl hydroxylase.

Inflammation has been identified as a significant contributor to hypoxia in various conditions, including Crohn’s disease, sepsis, and the no-reflow phenomenon evident in stroke^100,101^. The enzyme PHD plays a crucial role in connecting the signaling pathways for hypoxia and inflammation in a complex and intertwined manner^102^. Low oxygen levels can lead to the inactivation of PHD, which can trigger NF-κB activation, a primary inflammation regulator^102^. The resulting increase in pro-inflammatory leukocytes and cytokines can cause harm to vascular endothelial cells, impair vasoreactivity, and mitochondrial function, thus potentially worsening the state of hypoxia^11,102^. There exists a positive feedback loop between hypoxia and inflammation (Fig. 2).

#### Blood–brain barrier damage

Disruption of the BBB can lead to inflammation in specific areas of the brain, which in turn can initiate a series of pathological changes, including neurodegeneration and widespread inflammation^103^. When the BBB breaks down, T lymphocytes become activated in the periphery and then migrate across the endothelium into the brain, resulting in rapid clinical deterioration if the BBB is breached in a critical area of the nervous system^104^. The role of oxidative stress is frequently cited in the development of brain injury, with reactive oxygen species (ROS) and nitrogen species playing a key role in various mechanisms that contribute to the development of MS lesions^105–108^. The interaction of monocytes with brain endothelium can result in the production of ROS, leading to changes in TJs and cytoskeleton, BBB breakdown, and the entry of leukocytes into the CNS^109,110^. In the vascular spectrum of MS, the dysfunction of the BBB and the migration of activated leukocytes across the endothelial lining of blood vessels in the brain are some of the first abnormalities observed in the cerebrovascular system, and these abnormalities occur simultaneously with the release of cytokines and chemokines that promote inflammation^111^.

#### Hindered venous drainage

Extensive research has been conducted on the vascular characteristics associated with MS, and a specific MRI biomarker of MS called the ‘central vein sign’ has been proposed^112,113^. The cerebral vascular system, especially the veins, has been a focus of investigation in the early MS stages due to the occurrence of venous blood clots in the brains of MS patients^114^. In addition, MS patients have been observed to develop demyelination plaques around small and medium-sized veins in the CNS, along with perivascular infiltrations of inflammatory cells^114^. Multiple observations indicate that the vascular components play a role in the complex interplay of factors involved in the development and progression of MS, as well as the severity of the disease and the development of comorbidities^115–117^.

### Vascular intervention in MS

Venous intervention, or endovascular treatment, for MS, involves procedures designed to address CCSVI, a contentious theory suggesting that narrowed or blocked veins in the head and neck may have a contribution to MS development. The concept of venous intervention in MS, coined by Italian surgeon Zamboni, emerged from the hypothesis that improving blood flow in the veins draining the brain and spinal cord could potentially alleviate MS symptoms and slow down disease progression^118^. However, the validity of CCSVI as a substantial factor in the development of MS remains a topic of ongoing debate among scientists, and further research is necessary to establish its connection with MS. With his initial research lacking blinding and comparison groups, there is insufficient scientific evidence to support the association between CCSVI and MS, and the effectiveness of the surgery in alleviating MS symptoms remains inconclusive^119^.

Minimally invasive techniques, such as venous percutaneous transluminal angioplasty (PTA) or balloon angioplasty, more popularly known as ‘liberation therapy’ are commonly used for venous intervention in CCSVI associated with MS^117,120^. PTA involves threading a catheter through a vein to the narrowed or blocked area, followed by inflation of a balloon to widen the vein, and in certain instances, a stent may be inserted to maintain the vein’s patency^117,121^. Several independent studies have been unable to replicate the findings reported by Zamboni and his colleagues in their key studies^74,122–124^. Zamboni’s initial published research lacked blinding and a comparison group, and he did not disclose his financial ties to Esaote, the manufacturer of the ultrasound equipment used in CCSVI diagnosis^119,125^.

Various studies have found venous PTA to be safe but not effective as an intervention for patients with MS, and therefore cannot be recommended^72,117,126^. A study conducted in 2013 revealed that CCSVI occurs at a similar frequency in people with and without MS, and narrowing of cervical veins is equally prevalent in both groups^127,128^. Furthermore, there have been concerns regarding the safety of the venous intervention, with potential risks of complications such as vein damage, thrombosis, or restenosis^72,129,130^. The determination of whether to pursue venous intervention for MS necessitates a comprehensive assessment of the patient’s unique circumstances and medical background, and a careful consideration of the potential advantages and drawbacks^131^. It is important to remind patients that all reputable organizations and patient advocacy groups view vascular surgery for MS as an experimental approach^132^. Decision aids can assist patients in evaluating the risks and benefits associated with a particular medical intervention, enabling them to make informed decisions^132–135^. Recent reviews have indicated that there is no significant disparity in the prevalence of CCSVI between individuals with MS and those without and assert that, due to the withdrawal or termination of all ongoing trials, their updated review is conclusive and no further randomized clinical studies are necessary^117,128^.

## Conclusion

Despite considerable advances in immunology and molecular biology, the core cause of MS, including the triggers and pathways implicated in its development, remains unknown. Recent research reveals that vascular abnormalities play an important part in the genesis of MS, prompting the creation of a new classification called as vascular MS. Unlike other types of MS, vascular MS is caused by disease inside the blood arteries rather than by autoimmune processes. We aimed in this review to assess current data and give updates on genetics, pro-angiogenic variables, and vascular anomalies in the context of vascular MS, shining light on the significance of understanding the vascular aspects of MS.

## Ethical approval

Ethical approval was not required for this review.

## Consent

Informed consent was not required for this review.

## Sources of funding

No funding was obtained.

## Author contribution

P.P.: conceptualization, methodology, writing – original draft preparation, validation; A.S.: methodology, writing – original draft preparation; S.D.: writing – original draft preparation, review, and editing; P.I.: visualization, writing – original draft preparation; S.G.: writing – original draft preparation; A.S.: writing – original draft preparation; J.G.: visualization, writing – review and editing; M.K.: validation – writing, review, and editing; M.D.M.M.: visualization, writing – original draft preparation, review, and editing; S.K.: validation, writing – review and editing; A.D.M.M.: writing – review and editing; N.V.: writing – review and editing; O.A.H.: writing – review and editing.

## Conflicts of interest disclosure

The authors declare that they have no conflicts of interest.

## Research registration unique identifying number (UIN)

1. Name of the registry: not applicable.
2. Unique identifying number or registration ID: not applicable.
3. Hyperlink to your specific registration (must be publicly accessible and will be checked): not applicable.

## Guarantor

Priyadarshi Prajjwal and Pugazhendi Inban.

## Data availability statement

No data are available for this review.

## Provenance and peer review

Not commissioned, externally peer-reviewed.
